# Optimization of Thermal and Mechanical Properties of Polypropylene-Wollastonite Composite Drawn Fibers Based on Surface Response Analysis

**DOI:** 10.3390/polym14050924

**Published:** 2022-02-25

**Authors:** Konstantinos Leontiadis, Costas Tsioptsias, Stavros Messaritakis, Aikaterini Terzaki, Panagiotis Xidas, Kyriakos Mystikos, Evangelos Tzimpilis, Ioannis Tsivintzelis

**Affiliations:** 1 Department of Chemical Engineering, Aristotle University of Thessaloniki, GR-54124 Thessaloniki, Greece; leontiad@cheng.auth.gr (K.L.); tzimpi@auth.gr (E.T.); 2 Plastika Kritis S.A., R Street, Industrial Area of Heraklion, GR-71408 Heraklion, Greece; messaritakis@plastikakritis.com (S.M.); terzaki@plastikakritis.com (A.T.); 3 Thrace Nonwovens & Geosynthetics S.A., Magiko, GR-67100 Xanthi, Greece; pxidas@thraceplastics.gr (P.X.); kmystikos@thraceplastics.gr (K.M.)

**Keywords:** drawn polymer fibers, polymer composites, polypropylene, wollastonite

## Abstract

The thermal and mechanical properties of polypropylene-wollastonite composite drawn fibers were optimized via experiments selected with the Box-Behnken approach. The drawing ratio, the filler and the compatibilizer content were chosen as design variables, while the tensile strength, the melting enthalpy and the onset decomposition temperature were set as response variables. Drawn fibers with tensile strength up to 535 MPa were obtained. Results revealed that the drawing ratio is the most important factor for the enhancement of tensile strength, followed by the filler content. All the design variables slightly affected the melting temperature and the crystallinity of the matrix. Also, it was found that the addition of polypropylene grafted with maleic anhydride as compatibilizer has a multiple effect on the final properties, i.e., it induces the dispersion of both the antioxidant and the filler, tending to increase thermal stability and tensile strength, while, on the same time, deteriorates mechanical and thermal properties due to its lower molecular weight and thermal stability. Such behavior does not allow for simultaneous maximization of thermal stability and tensile strength. Optimization based on a compromise, i.e., targeting maximization of tensile strength and onset decomposition temperature higher than 300 °C, yields high desirability values and predictions in excellent agreement with verification experiments.

## 1. Introduction

Polypropylene (PP) is one of the most popular thermoplastics. Globally, it presents the second highest production volume, after polyethylene (PE). Consequently, significant research effort is performed towards the enhancement of its thermal, mechanical, electrical and other properties. In this direction, it is a common practice to utilize inorganic fillers for producing PP composite polymer matrices. Polypropylene is used in various forms such as solid parts, films, and sheets. However, almost one third of its production refers to the form of fibers [1], which present a wide spectrum of both conventional and modern industrial applications, such as the development of fibrous porous media for waste water treatment, textiles, petroleum engineering, thermal insulation etc. [2,3].

Wollastonite (CaSiO_3_) mineral is commonly found as micro-sized needle like particles, although nano-sized synthetic wollastonite particles have been produced [4]. It is commonly used as a filler for producing PP composite materials that present enhanced mechanical properties [4,5,6,7,8,9,10,11], higher crystallinity [5,7,8] and higher thermal stability [3,12]. Although, PP is the most studied polymer for producing wollastonite composites [13], there are only few studies for PP-wollastonite composite fibers and especially drawn fibers [14]. The biaxial drawing of polymer films and the uniaxial drawing of polymer fibers aligns macromolecular chains [14,15,16,17,18,19,20,21,22], resulting in a significant increase of tensile strength [18,19,21,22,23,24]. Besides polymer chain alignment, drawing can also induce the alignment of needle like particles to the drawing axis, improving the mechanical stress transfer from the polymer matrix to the filler [18,19,25,26]. However, in most cases, the increase of tensile strength through the macromolecular chain alignment is tremendous (for example the use of a drawing ratio equal to 7 can increase more than ten times the PP’s tensile strength, from around 30–40 MPa to around 400–500 MPa) [14,27]. Regarding the drawn PP fibers, various fillers have been implemented, which can be classified in three main categories, i.e., carbonaceous fillers, mineral clays and other inorganic nanoparticles [27]. The first category includes graphene nanoplatelets [28,29], multi- or single- wall carbon nanotubes [14,19,30,31,32,33,34,35,36,37,38,39,40], or carbon nanofibers [18,41]. Mineral clay fillers include montmorillonite, either modified [42,43,44,45,46], or not [42,46], synthetic modified hectorite [47], modified hydrotalcite [44], boehmite [48] and wollastonite [14]. Other inorganic nanoparticles that were implemented include TiO_2_ [49], BaSO_4_ [20], cobalt phthalocyanine [50], fumed silica [51,52] and ZnO [53]. More details about nanocomposite PP fibers can be found in a recent review study [27].

PP, as a polyolefin, presents a non-polar hydrophobic nature, in contrast with the polar and hydrophilic nature of most inorganic fillers. A common approach to induce thermodynamically favorable intermolecular interactions and, thus, improve the affinity between the polymer matrix and the filler, is to modify/functionalize the PP matrix and/or the additive. Modifications can be applied to the PP matrix, to the filler or to both. The most common practice is the use of PP grafted with maleic anhydrite (PP-g-MA) as compatibilizer [6,14,19,43,44,46,47,52]. Considering wollastonite particles, several surface modifications have been studied. For example, pimelic acid was used as surfactant to increase PP-filler adhesion [7] and induce the nucleation of *β*-PP crystallites [9,54,55,56,57,58]. Stearic acid was also investigated as a wollastonite modifier [59,60] resulting in better filler dispersion [59]. Furthermore, the use malonic acid in wollastonite treatment was found to induce *β*-nucleation of the PP matrix, resulting in better filler dispersion and improved mechanical properties [10]. Liang improved the polymer/wollastonite affinity using PP-g-MA as compatibilizer and through filler modification using betaine as coupling agent [11]. The latter surface treatment of the mineral resulted in composites with better thermal stability [12].

Besides the filler type, the filler size and shape are crucial factors for improving mechanical properties. Compared to micro-sized fillers, the nano-sized ones are preferred as they offer significant improvement at relatively low filler content, without disturbing the production process [13,61]. 

In our previous preliminary study, we have investigated the use of various fillers, such as talc in two particle sizes, single wall carbon nanotubes, wollastonite and attapulgite, in PP drawn fibers under a constant drawing ratio equal to 7 [14]. It was found that needle like fillers, such as wollastonite and carbon nanotubes exhibit more promising results considering the improvement of mechanical and thermal properties [14]. Also, in that work [14], it was found that multiple synergistic and competitive phenomena take place due to the presence of other, different than the filler, additives (i.e., antioxidant and compatibilizer). For example, although the used compatibilizer was mainly added for enhancing the dispersion of the filler, it was found that it primarily enhances the dispersion of the antioxidant, increasing the thermal protection, which, in turn, leads to improved properties [14]. However, at high compatibilizer contents, the low molecular weight of the compatibilizer may have a negative impact on the drawing process, and, thus, its beneficial effect on mechanical strength may be reversed. In addition, the use of such additives, commonly used in industrial practice, besides the above mentioned effect on the materials’ properties, also affect the production process. For example, the low molecular weight of the additives results in fibers of larger diameter, due to the higher melt flow index, compared to samples with no additives, under the same processing conditions. In general, materials’ devolvement under laboratory and industrial conditions can be quite different, e.g., in laboratory samples, purity is of primary importance in order to study the material’s behavior and properties. On the contrary in real-life applications, that is in industrial samples, the addition of various additives, such as antioxidant, coloring substances, UV protection agents etc., is a common practice. However, the effect of such additives on the properties of the final material, cannot be easily evaluated and optimized by the industries for practical reasons. Thus, the fundamental understanding of the interactions and the effect of such common industrial additives is of great practical importance, both for optimizing currently adopted processes, but also for developing more suitable and application-specific additives. However, due to multiple and complex effects, like the above mentioned, optimization of processes cannot be easily accomplished through single variable alteration experiments (i.e., changing only one variable each time and keeping all other variables constant).

In this direction, surface response methodology is a well-established approach for optimizing processes, e.g., finding the values of the independent (design) variables that maximize a response variable of interest. More specifically, surface response methodology is a set of various mathematical/statistical procedures for the optimization of response variables [62]. Thus, response surface methodology has attracted interest in various Chemical Engineering processes such as materials’ development [63,64], wastewater treatment [65] etc. A plot of the response variable versus two independent variables is the graphical representation of the response surface [62]. The Box-Behnken design is a methodology for modelling (fitting) response surfaces [62]. It is a three level design approach and its main advantage is the rather low number of required experiments. A disadvantage of Box-Behnken design is that, since it is a spherical design, it is not preferable in cases in which the region of interest is cuboidal [62]. 

In this work, we studied the optimization of the mechanical and thermal properties of polypropylene/wollastonite composite drawn fibers that also contain common industrial additives (antioxidant and compatibilizer). The Box-Behnken design was adopted in order to perform the surface response analysis. With the exception of our recent previous work [14] for PP composite drawn fibers with various fillers, including preliminary experiments with wollastonite, the development of PP-wollastonite drawn fibers has not been studied (wollastonite has been widely used as filler for PP, but not for the production of drawn fibers). Besides this, the use of other additives that exhibit multiple effects and the process optimization through surface response methodology contribute to the novelty and significance of the current work.

## 2. Experimental

### 2.1. Materials

In all cases, isotactic polypropylene was mixed with masterbatches containing the used additives, i.e., wollastonite, compatibilizer and antioxidant. The most important characteristics of the used materials are shown in Table 1. 

### 2.2. Experimental Apparatus and Procedure

The quantities of each masterbatch required for the preparation of a composite material were weighted in a laboratory balance (KERN PLS 1200-3A) with an accuracy of 0.001g. Eight composites were prepared. However, from these composites, a total of fifteen fiber samples were obtained, since some of them were drawn at two different ratios (more information are given in the “Design of Experiments” section). All composites contained 4% wt. of the antioxidant masterbatch that corresponds to 0.82% wt. of the active antioxidant compound. The percentage composition of the eight investigated composites is presented in Table 2. 

After weighing, each mixture of masterbatches was introduced in a twin-screw extruder (HAAKE Rheodrive 5001) with a nozzle diameter of 3 mm. The temperature profile was the same for all the samples. In more detail, the temperatures of the four heating zones, from the feeding stage to the outlet nozzle, were set equal to 190, 210, 215 and 220 °C, respectively, while the motor speed was set equal to 25 rpm. This initial stage was incorporated to ensure adequate mixing of masterbatches. The polymer melt exiting the extruder was immersed in a water bath (~ 15 °C) to solidify. The produced filaments had a diameter of 1–2 mm.

Subsequently, the produced filaments were cut into pellets (2–3 mm length) and introduced in a single-screw extruder (Noztek Xcalibur) with a nozzle diameter of 1.6 mm. The temperature of the three heating zones from the feeding stage to the nozzle were set equal to 215, 225 and 210 °C, while the motor speed was set to 15 rpm. After exiting the extruder, the polymer melt was cooled by an air fan (the distance from the nozzle to the fan was around 2 cm) and then it was immersed in a water bath (~15 °C). After the water bath, the produced filament was collected automatically by a winding device (Noztek filament winder). The produced filaments exhibited a diameter of 0.4–0.7 mm and were used for the subsequent step of solid-state drawing.

**Figure 1:**
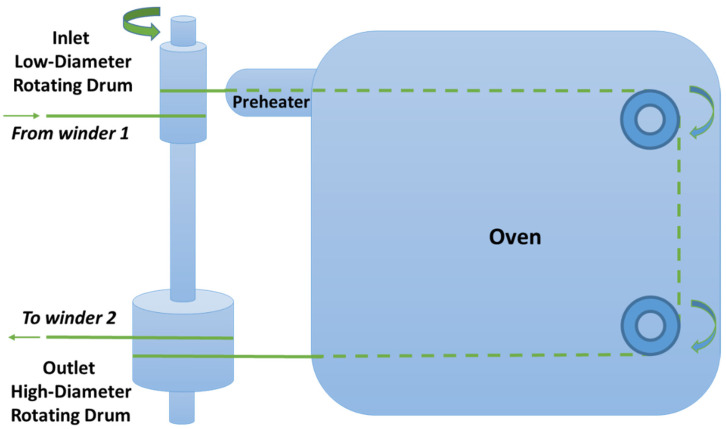
Scheme of the experimental drawing apparatus.

The drawing apparatus is presented in Figure 1. The filament, after the first winder, is winded twice in the low-diameter inlet drum, passes through a small preheating zone, and enters in an oven of constant temperature. After exiting the oven, it is winded twice in the high-diameter outlet drum and collected by the second winder. The temperature of the preheater was equal to 120 ± 2 °C, while the temperature of the oven was equal to 140 ± 1 °C. Such temperatures are below the melting point of the composites, which in all cases ranged between 162 and 168 °C. 

The inlet and outlet drums are attached to the same axis and, consequently, rotate at the same speed (5 rpm in this study). In this way the drawing ratio (*λ*) is equal to the ratio of their diameters. The apparatus allows the exchange of the outlet rotating drum, with drums of various diameters, thus achieving the required drawing ratio. Three drawing ratios were used for the experiments (5, 7 and 9). The diameter of first inlet drum was equal to 20 mm. Consequently, an outlet drum with diameter equal to 100 mm and another one with diameter equal to 140 mm were used to obtain drawing ratios equal to 5 and 7, respectively. In order to achieve a drawing ratio equal to 9, two subsequent drawings with a draw ratio equal to 3 were performed, using an outlet drum of 60 mm diameter. 

Typically, 5–15 m of drawn fibers were produced. After drawing, the produced fiber diameter ranged from 0.13 to 0.31 mm, depending on the drawing ratio and the diameter of the initial filaments. To ensure homogeneous drawing, each filament was marked every 50 mm prior to the drawing process. At the end of the drawing process, only the sections with marks at a distance of 250 ± 10 mm, 350 ± 10 mm and 450 ± 10 mm, for actual drawing ratios of 5.0 ± 0.2, 7.0 ± 0.2 and 9.0 ± 0.2, respectively, were used for characterization experiments. By using this approach, it is ensured that the filaments used for characterization have undergone the desired degree of drawing, independently of their initial diameter. Also, the above mentioned range of fiber diameters covers all drawing ratios and samples, while the diameters within the same sample present similar values, as shown for two characteristic samples (one prepared using drawing ratio equal to 7 and one using drawing ratio equal to 9) in Table S1 of the Supplementary Information file. As it can be observed, the ten different pieces that were used for the tensile tests of sample DOE 3 (see Section 3 for more details) exhibited an average value (±standard deviation) equal to 0.23 ± 0.01 mm and the respective value for sample DOE 11 was 0.16 ± 0.01 mm. The difference in the final diameter of different samples arises from the different drawing ratios (5, 7 and 9), as well as from the fact that the final extrusion was performed under the same speed (same rpm), but with samples of different composition. For example, sample DOE 3 contained 15% of compatibilizer (that exhibits a high melt flow index in order to allow for easy mixing), while sample DOE 11 contained no compatibilizer. Thus, the latter flows slower and, consequently, for the same extrusion speed, less polymer exits from the extruder, rendering the produced filament of smaller diameter. Thus, variables such as the compatibilizer content have multiple effects both on the physicochemical aspects and the process parameters. By the response surface analysis (see Section 3 for more details), the overall effect of such variables can be evaluated.

### 2.3. Characterization

The thermal properties of the drawn fibers were examined with differential scanning calorimetry (DSC) and thermogravimetric analysis (TGA) using a Shimadzu DSC-50 and a Shimadzu TGA-50 apparatus, respectively. The DSC measurements were performed in the temperature range of 40–230 °C, under nitrogen atmosphere (flow rate of 20 mL min^−1^), and with a heating rate of 10 °C min^−1^. The mass of the samples for the DSC measurements was weighed using a Sartorius B120s scale (of 0.0001 g accuracy). The TGA measurements were performed in the temperature range of 40–450 °C, in air atmosphere, with a heating rate of 20 °C min^−1^. The samples for the DSC and TGA measurements were selected from random pieces of the drawn fibers. 

Tensile tests were carried out using a Hans Schmidt & Co GmbH Universal Testing Machine ZPM equipped with a Pacific PA6110 loadcell. Head speed was set equal to 100 mm min^−1^. The tensile strength, Young’s modulus and elongation at break values were obtained by the average value of 8–12 repetitions for each measurement using random pieces of the drawn fibers. 

## 3. Design of Experiments

**Figure 2:**
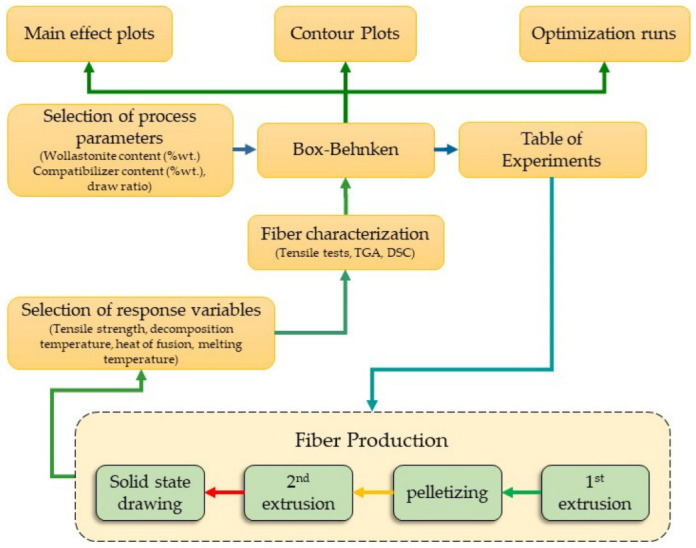
Flow chart of experimental and optimization procedure.

Optimization of the properties of the composite drawn fibers was carried out using a surface response methodology, through design of experiments (DOE) with the Box-Behnken method. The Minitab^®^ 20.4 software was used. A flow chart of the experimental and optimization procedure is presented in Figure 2. Initially, the independent (design) variables are selected along with their upper and lower limits, e.g., for the drawing ratio the lower limit was 5 and the upper limit was 9. Then, from these input data and through the Box-Behnken design, the required experiments are extracted (see Table 3). After the execution of the required experiments, the samples are characterized (determination of the response variables). The values of the response variables are then inserted in the Box-Behnken design and the required modelling is executed to yield the main effect and contour plots. Finally, from such data the optimization is carried out.

The wollastonite content (on an inorganic filler base), the compatibilizer content (on a masterbatch base) and the drawing ratio were chosen as design variables. The tensile strength at break, the melting temperature, the heat of fusion and the onset decomposition temperature (defined as the temperature at which 3% wt. mass loss occurs) were set as response variables. The values of the design parameters for each experiment, as derived by the Box-Behnken method, are presented in Table 3. Here it is worth mentioning that the last three experiments, shown in Table 3, are three independent repetitions of the same experiment. In this way, all errors in the measurement of properties and the processing conditions are effectively accounted for in the optimization procedure shown in the next sections.

## 4. Results and Discussion

### 4.1. Single Design Variable Effect on Response Variables

The results of all the fifteen DOE experiments, and more specifically the tensile strength at break (*TS*), the elastic modulus (*EM*), the elongation at break (*EatB*), the onset decomposition temperature (*T_dec_*), the melting temperature (*T_m_*) and the heat of fusion (Δ*H*) are presented in Table 4. The onset decomposition temperature was set as the temperature for which the remaining mass is 97% of the initial mass of the composite material (3% wt. mass loss). 

Initially let us shortly discuss the results presented in Table 4. Among the various samples, different property values can be observed. The reported differences range from negligible to considerable. For example, significant differences were observed in the tensile strength (the lowest observed value was 291 MPa for DOE 4, while the highest one was almost the double, equal to 535 MPa for sample DOE 11). Similar differences can be observed also for the elastic modulus. A careful examination of the results reveals a mild pattern between tensile strength/elastic modulus and elongation at break. Samples with high tensile strength exhibit low elongation at break. Also, from the results of Table 4 it can be concluded that the same degree of drawing does not have the same effect on different composites. This arises from their different composition, e.g., it is widely known that addition of inorganic fillers in polymers usually causes a decrease in elongation at break. Thus, besides the different drawing ratio, the different composition of the samples is responsible for the different effect of drawing. Considerable differences are also observed in the decomposition initiation temperature. Such differences can be attributed to the relative amounts of compatibilizer and antioxidant and their interactions [14] and also to the presence of wollastonite. On the other hand, less important differences are observed in the heat of fusion and especially in the melting temperature. From this observation it can be concluded that the crystallinity of the fibers is mainly governed by the drawing process and it is less influenced by the additives.

**Figure 3:**
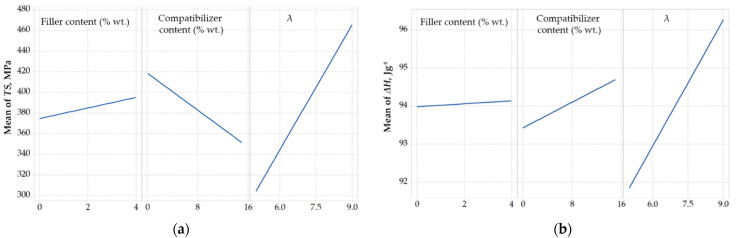

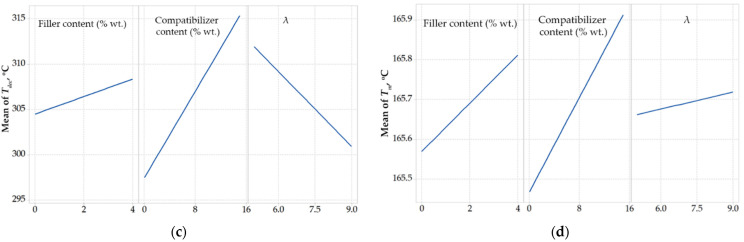
Main effect plots for (**a**) tensile strength (*TS*), (**b**) heat of Fusion (Δ*H*), (**c**) onset decomposition temperature (*T_dec_*), and (**d**) melting temperature (*T_m_*).

The effect of design variables on the response variables is illustrated in Figure 3. As shown in Figure 3a, the drawing ratio is the factor, among the three design variables, with the most significant effect on tensile strength, which is in agreement with the findings of Fambri et al. [52]. Also, it is observed that the tensile strength increases to a small extent with increase in wollastonite content. Such rather mild improvement indicates a weak interaction between the filler and the polymer matrix. Finally, the non-negligible decrease of tensile strength with increase in compatibilizer content is in agreement with literature and can be attributed to the lower molecular weight of the compatibilizer, i.e., during drawing, the shorter chains are overstretched and, consequently, hinder the improvement of mechanical properties [66]. 

Similarly, to the tensile strength, the heat of fusion (Figure 3b) is mostly affected by the drawing ratio. However, such effect is rather small, since, as shown in Figure 3b, in the studied drawing ratio range of 5 to 9, the heat of fusion increases only by 4%. This is in agreement with literature, since it is known that the stretching increases crystallinity and favors the transformation of *β*-PP crystals to *α*-PP crystals [67]. It seems that the drawing ratio of 5 is enough to induce crystal formation and, thus, the heat of fusion of such samples is similar to those drawn at the ratio of 7 or 9. However it seems not enough to cause adequate alignment of crystal regions and, thus, the tensile strength of samples drawn at a ratio equal to 5 is considerably lower that the tensile strength of samples drawn at higher ratios. Moreover, both the filler and compatibilizer content have a negligible effect on melting enthalpy, i.e., alter the melting enthalpy less than 1 J g^−1^ in all cases, suggesting that wollastonite does not exhibit any considerable nucleation ability for PP. It has been reported that *α*-PP is formed in the presence of wollastonite, while only after suitable modification wollastonite can exhibit strong nucleation ability for *β*-PP crystals [54]. 

As shown in Figure 3c, increasing the drawing ratio decreases the onset decomposition temperature, which can be explained by the smaller diameter of the fibers and the consequent faster heat transport to the material. Regarding the effect of the compatibilizer on the onset decomposition temperature, a steep increase is observed. This is in agreement with our previous findings [14] regarding the strong interactions between the phenolic antioxidant and the compatibilizer that result in improved dispersion of the antioxidant and, thus, increase thermal protection. This increase of thermal protection leads to less polymer decomposition during processing and, thus, the use of the compatibilizer, although directly has a negative effect on tensile strength, indirectly and under some specific conditions, may lead to improved mechanical strength. Such favorable compatibilizer-antioxidant interactions may act competitively to the compatibilizer-filler interactions and provide an explanation for the mild increase of the onset decomposition temperature that is observed with increase in wollastonite content. 

Lastly, the melting temperature (Figure 3d) does not seem to be affected by the design variables and very small changes, lower than 1 °C in all cases, were observed.

### 4.2. Combined Effect of Design Variables on Response Variables

Melting temperature is not further considered in the optimization procedure, since, as discussed above, it is not significantly affected by changing the design variables in the studied range. 

**Figure 4:**
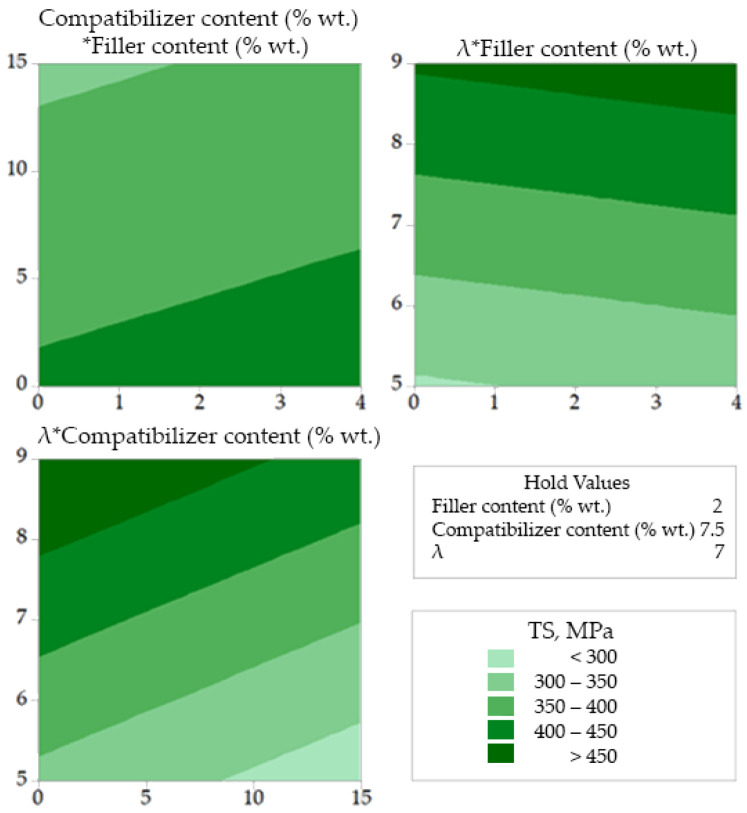
Contour plots for Tensile strength correlations.

**Figure 5:**
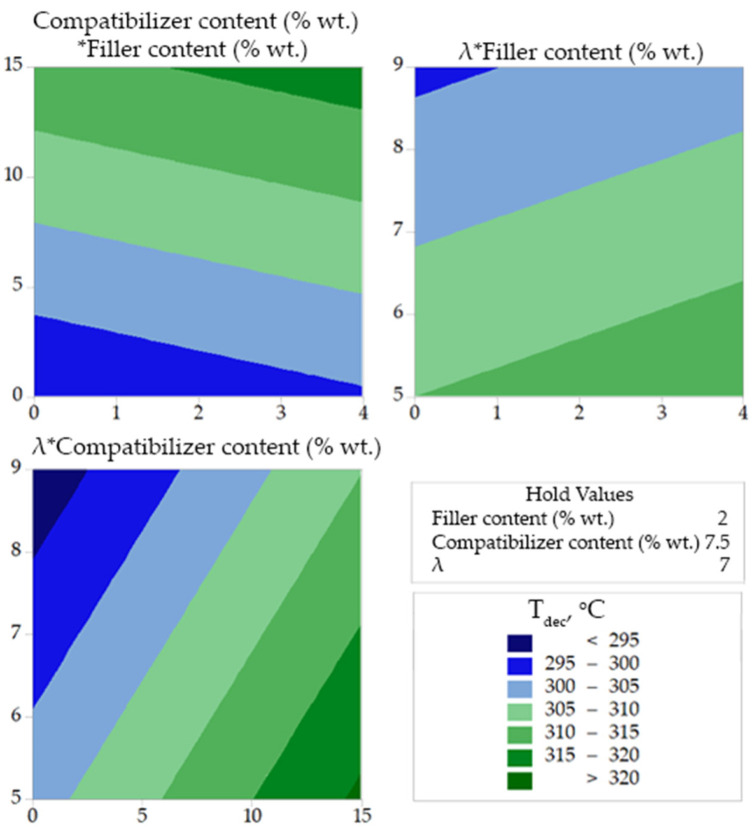
Contour plots for decomposition temperature correlations.

**Figure 6:**
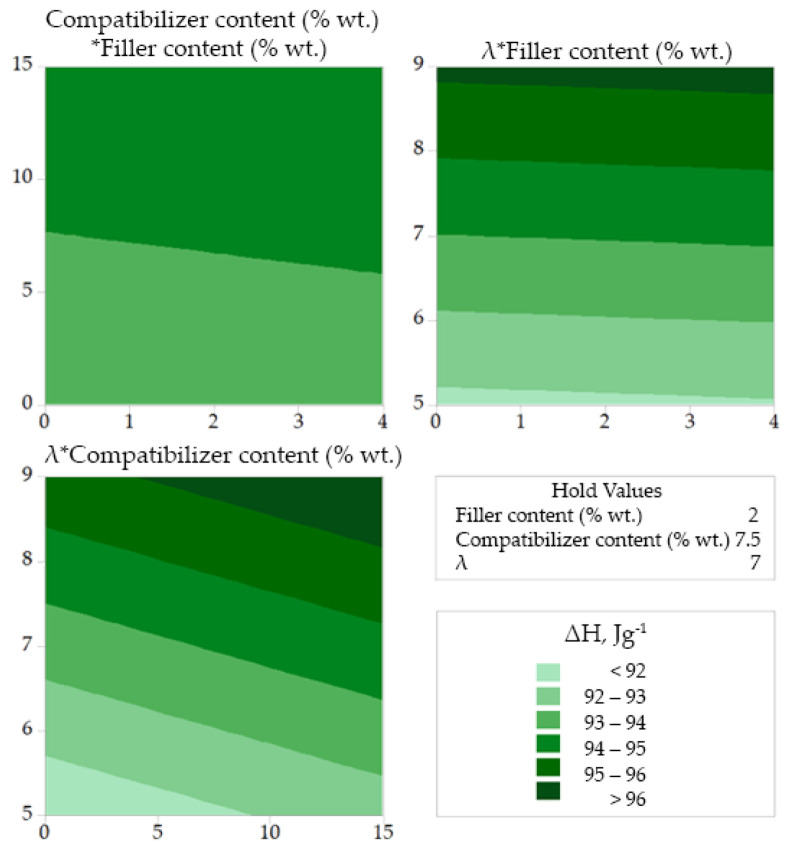
Contour plots for melting enthalpy correlations.

In Figure 4, Figure 5 and Figure 6, the combined effect of design variables (in pairs and for a fixed value of the third design variable) on tensile strength, onset decomposition temperature and heat of fusion are presented as contour plots. In each of these plots the fixed (hold) value of the relevant design parameter is shown in the right part of the figure. For example, in Figure 4 the hold value for the filler content is 2% wt. and corresponds to the bottom left plot (with the title λ*Compatibilizer content). In each plot, the values of the first response variable (e.g., *λ* in the λ*Compatibilizer content plot) appearing in the title, correspond to the *Y*-axis, while the values of the second response variable (e.g., *Compatibilizer content* in the λ*Compatibilizer content plot) correspond to the *X*-axis. From this plot (down left in Figure 4), it is revealed that for compatibilizer content higher than 10% wt. and for a fixed wollastonite content of 2% wt. there is no drawing ratio (in the studied range) that can produce fibers with tensile strength higher than 450 MPa. Also, as shown in Figure 4 (upper left plot), for a fixed drawing ratio equal to 7, the tensile strength increases by increasing the filler content. From the upper right plot of Figure 4, it becomes evident that for a compatibilizer content equal to 7.5% wt., very high values of drawing ratio (>8.5) are needed to achieve tensile strength higher than 450 MPa. 

The increase of thermal stability due to compatibilizer-antioxidant interactions, which were already mentioned in the previous section and were reported in our previous work [14], is clearly detectable in Figure 5. It is clear that the onset decomposition temperature, *T_dec_*, increases with increase in the compatibilizer content (see upper left plot and down left plot of Figure 5). Also, the mild positive effect of the filler on the onset decomposition temperature, which was concluded from the main effect plots (Figure 3), is also revealed by the contour plots (Figure 5). More precisely, as shown in the upper left plot of Figure 4, the addition of filler has a minor positive effect on *T_dec_* for a constant value (equal to 7) of the drawing ratio. A similar mild positive effect of the addition of filler on *T_dec_* is revealed by the upper right plot of Figure 4 for a fixed value of the compatibilizer content, equal to 7.5% wt. 

Finally, from Figure 6 it can be concluded that the drawing ratio has more pronounced effect on the heat of fusion (which is related to the degree of crystallinity of the fibers), while any influence of the filler and compatibilizer content is rather small.

Similar plots can be constructed for any desired hold value of the three design variables. In general, there is high amount of information that can be extracted from such plots, that makes the interpretation time consuming and difficult. However, the available information from the Box-Behnken analysis can be utilized for optimization purposes. A first approach for the optimization is based on the overlapping areas of the contour plots, for desired values of the response variables, as presented in the next section.

### 4.3. Optimization Based on Overlaid Contour plots

**Figure 7:**
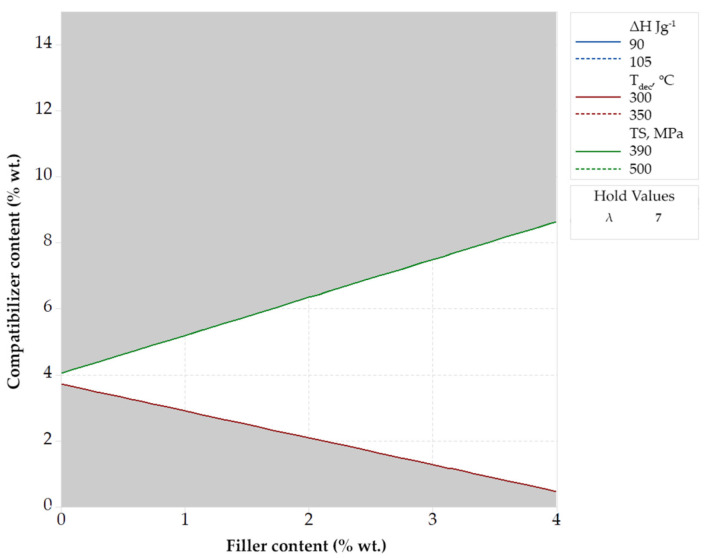
Optimization based on the combination (overlapping) of contour plots for specified ranges of Δ*H*, *T_dec_* and *TS* using a hold value for the drawing ratio equal to 7.

In Figure 7, a combined contour plot is presented using a hold value equal to 7 for the drawing ratio. The white area in Figure 7 represents the range of values for filler and compatibilizer content that can be used to achieve tensile strength in the range of 390–500 MPa, decomposition temperature in the range of 300–350 °C and melting enthalpy in the range of 90–105 J g^−1^. Such ranges were selected since they correspond to improved tensile strength (*TS*), thermal stability (*T_dec_*) and crystallinity (as indicated by the enthalpy of fusion) compared to the values of neat PP.

It can be observed (Figure 7) that, for compatibilizer content equal to 4% wt. the produced fibers present *TS*, *T_dec_* and Δ*H* values within the specified ranges, independently of the wollastonite content, even for the polymer matrix with no filler. As it is shown in our previous study, this can be attributed to the fact that the addition of compatibilizer improves the dispersion and, thus, the protective action, of the antioxidant enhancing, indirectly, the thermal stability [14]. The observed tensile strength is simultaneously improved since the enhanced protective action of the antioxidant results in less decomposition during thermal processing [14]. 

From the same plot (Figure 7) it is revealed that upon increasing the compatibilizer content at values higher than 4% wt., more filler is required to achieve the target values of the response variables. This is a rather unexpected observation that can be explained by the multiple action of the compatibilizer. More specifically, the addition of the compatibilizer tends to increase the thermal stability and the tensile strength by enhancing the dispersion of both the filler and, as mentioned above, the antioxidant [14]. On the same time, its low molecular weight has a negative effect on the stretching potential and tends to decrease the tensile strength (as it was depicted by the results presented in Figure 3a). The net effect of such phenomena results in the behavior presented in Figure 3a,c, i.e., increasing the compatibilizer content in composite materials tends to increase the thermal stability (see Figure 3c) and decrease the tensile strength (see Figure 3a). To counterbalance such deterioration of tensile strength, the wollastonite content must be increased.

If stricter criteria are used for the desired values of *TS* (e.g., 500–550 MPa) and *T_dec_* (e.g., 320–350 °C), then such dual effect of compatibilizer becomes more intense and the desired properties cannot be reached even by using a high drawing ratio, equal to 8.2 (see Figure S1 of the Supplementary Information file). If instead, broader ranges are defined they can be easily reached for a wide range of compatibilizer and wollastonite content (see Figure S2 of the Supplementary Information file). Similar plots can be obtained by setting hold values for the other two design variables (see the Supplementary Information file, Figures S3 and S4) for some representative plots assuming constant wollastonite content, equal to 2% wt., and constant compatibilizer content, equal to 7.5% wt., respectively. Such optimization approach is versatile and extremely useful when a single design variable can be fixed and a rather wide range of the response variables (properties) can be accepted. 

However, a drawback of this approach is that the obtained plots inform only for the fulfillment, or not, of the optimization criteria and do not show any information about the specific values of the response variables (properties). For example, in the case presented in Figure 7, the filler and the compatibilizer content that correspond to the white region, yield values of tensile strength between 395 and 500 MPa, however, no information is obtained about the specific value of tensile strength in the two-dimensional (white in that plot) area. To overcome this drawback, an optimization can be performed based on: (1) qualitative criteria (e.g., maximizing the value of one or more response variables), (2) specific target values and not ranges of values for the response properties and (3) a combination of the above. In addition, such optimization routes can be performed without fixing the value of any of the design variables, as presented in the next section.

### 4.4. Optimization Based on Specific Target Values

**Figure 8:**
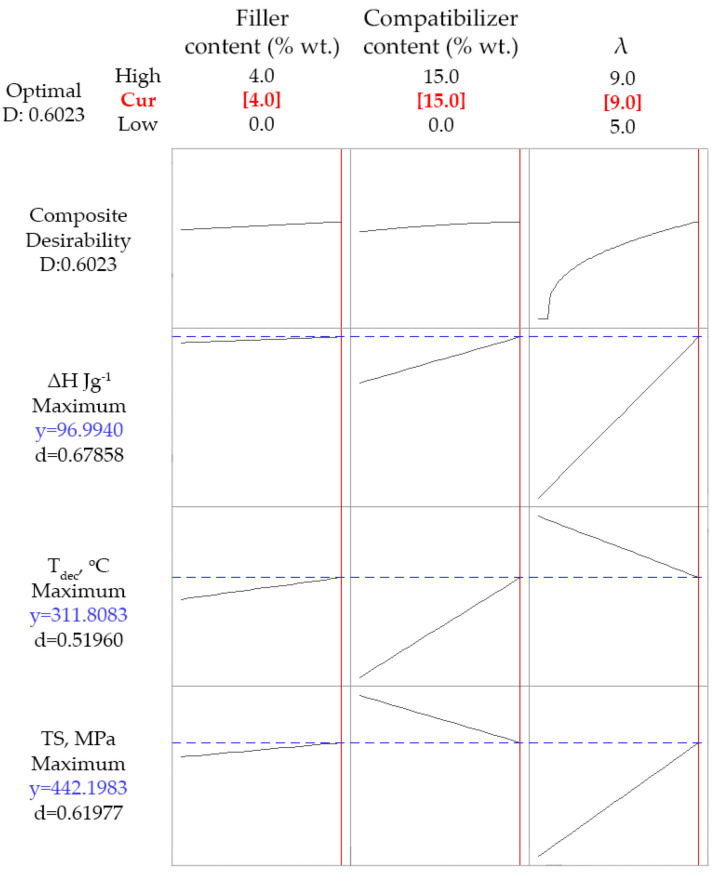
Optimization run targeting maximization of all the three response variables (*T_dec_*, Δ*H* and *TS*). Red values (over the graphs) represent the optimized values of the design variables, while blue values (on the left of the graphs) represent the obtained (correlated) values of the response variables.

In Figure 8, the optimization results regarding the simultaneous maximization of Δ*H*, *T_dec_* and *TS*, are presented. In the upper part of the plot, the optimum values for each one of the design variables are presented with red. In the left part of the plot the overall desirability (*D*) and the partial desirability component (*d*) for each one of the response variables are presented. In general, the desirability is equal to unity if the maximization criterion is fully fulfilled, e.g., the value of *d* for *TS* would be unity if the obtained value of such property would have been equal to the maximum value that was measured in the 15 experiments (535 MPa see Table 3). However, as can be seen in Figure 8, the overall and the partial desirabilities are rather low, with the latter ones ranging between 0.52 and 0.68. Such low values indicate that all three maximization criteria cannot be simultaneously fulfilled. In the obtained composite materials, simultaneous maximization of Δ*H*, *T_dec_* and *TS* cannot be obtained, due to the multiple effect of the compatibilizer that was mentioned in the previous section, i.e., its addition tends to increase *T_dec_*, but to decrease *TS*. 

Furthermore, if Δ*H* is excluded from the optimization procedure and only the other two criteria are used (maximization of both *T_dec_* and *TS*), no substantial difference is observed (see Figure S5 of the Supplementary Information file). In other words, the model cannot find values for the design variables that maximize both response variables. This, can be mainly attributed to the dual effect of the compatibilizer. Having in mind its low thermal stability [14] and its, relatively to the used PP matrix, lower molecular weight (see MFI in Table 1), it is reasonable to expect the deterioration of both the thermal stability and the tensile strength upon increasing its content. On the other hand, the addition of the compatibilizer favors the dispersion of the filler and the antioxidant (as reported in our previous study [14]) tending to indirectly increase both thermal stability and mechanical properties. The net effect of such phenomena is the decrease of tensile strength, shown in Figure 3a, and the increase of the onset decomposition temperature, shown in Figure 3c, upon increasing the compatibilizer content. Consequently, since the one response variable increases and the other one decreases with the addition of compatiblizer, there is no way of maximizing both of them.

Nevertheless, if only the maximization of *TS* is required, the optimization procedure results in maximum values for both the filler content (4% wt.) and the drawing ratio (9), as well as minimum compatibilizer content (0%). The desirability in this case is increased to 0.89 (see Figure S6 of the Supplementary Information file). 

**Figure 9:**
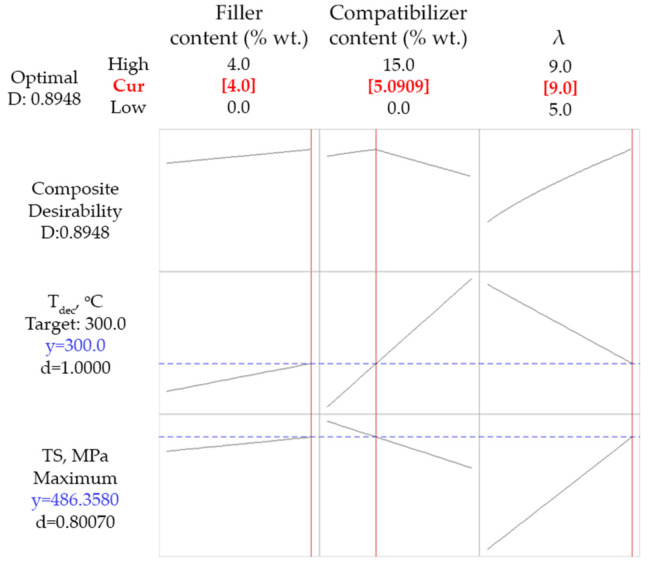
Optimization run targeting maximization of tensile strength (*TS*) and decomposition temperature (*T_dec_*) higher than 300 °C. Red values (over the graphs) represent the optimized values of the design variables, while blue values (on the left of the graphs) represent the obtained (correlated) values of the response variables.

Finally, since simultaneous maximization of *T_dec_* and *TS* cannot be accomplished due to the multiple effect of the compatibilizer, and in order to find a compromise, the optimization was performed by setting as criteria the maximization of *TS* and a lower limit for *T_dec_* (equal to 300 °C). An onset decomposition temperature equal to 300 °C can be considered as an acceptable increase of thermal stability (the neat PP sample exhibits a *T_dec_* of 271 °C). The results of this optimization run are presented in Figure 9. The obtained desirability values are 1 for the decomposition temperature and 0.8 for tensile strength, while the overall desirability is fairly high (about 0.9), meaning that the decomposition temperature criterion is fully met, while the tensile strength criterion is almost met. The predicted/optimum values of the design variables for such case are 4% wt. wollastonite content, 5.1% wt. compatibilizer content and drawing ratio equal to 9.

After the latter optimization, a sample with the aforementioned composition and drawing ratio was produced, following the process described in the experimental section. Experimental tensile test and TGA results are shown in Table 5. It is shown that optimization predictions and experimental results are in a very good agreement with each other as revealed by the deviations of the optimization results from the experimental values (around 2% for tensile strength and lower than 1% for the onset decomposition temperature).

Here, it is should be stretched, that some of the accomplished values for tensile strength, e.g., values higher than 450 MPa, are comparable to the respective values of various carbon and low-alloy steels [68]. The different molecular weight of PP, the different additives and the different processing (e.g., different apparatus for drawing or only one extrusion instead of the two extrusions used in this study) hinder the comparison of the presented results with other literature data. Although, there are no data for PP/wollastonite composite drawn fibers in the literature, comparisons can be made with other fillers. In any case, it is worth mentioning that the tensile strength values reported in the present study are higher than respective values (123–178 MPa) of drawn composite PP fibers with hydrotalcite and nanoclays [44], while they are similar with reported values (479 MPa) for PP/carbon-nanofiber nanocomposite drawn fibers [41]. Composite PP fibers, drawn at a higher ratio and containing needle-like fillers, such as multi wall carbon nanotubes (with lower filler content equal to 0.5% wt.), exhibit similar results for tensile strength (416 MPa) [19]. Comparable results were obtained with the values reported for composite PP/fumed silica drawn fibers with 2% wt. filler concentration (346 MPa), which were drawn at the same ratio with the one used in this study (383 MPa—see DoE sample 9) [52]. However, the cost of wollastonite is much lower than the cost of carbon nanofibers or multi wall carbon nanotubes. Also, the two extrusions used in this study may be avoided, and thus the thermal oxidation/decomposition and the related deterioration of mechanical properties can also be avoided. Consequently, even higher values for the tensile strength are possible. Finally, it is worth mentioning that one of the highest tensile strength values, ever reported for PP composite drawn fibers, is 860 MPa and was achieved by the addition of montmorillonite and ultra-high drawing ratio equal to 27 [69], which, however, results in non-negligible shrinkage of fibers with aging.

Finally, it is worth briefly discussing the possible extension of some observations of this study to other types of fibers and materials. Firstly, no matter the used polymer, the impact of drawing on the tensile strength of fibers is severe and renders the effect of the filler and the compatibilizer of lower importance. Also, the inability of simultaneous maximization of thermal and tensile strength is expected to occur in various other (non PP) fibers, in which low molecular weight additives are used. Moreover, similar issues are expected to be present in other forms of drawn materials, e.g., in biaxial drawn films. Furthermore, in non-drawn samples the synergistic effect due to compatibilizer antioxidant interactions is expected to positively influence the properties of the composites, since the effect of the low molecular weight of such additives is not significant if drawing is not applied. However, similar issues and multiple competitive and/or synergistic effects may arise from other common industrial additives, such as substances for UV protection, or coloring agents.

## 5. Conclusions

Polypropylene–wollastonite composite drawn fibers were produced using PP grafted with maleic anhydrite as compatibilizer and a combination of phosphite and phenolic type antioxidants. It was observed that many of the produced samples exhibited values of tensile strength higher than 450 MPa, which is a remarkable increase compared to the strength of neat polypropylene (around 30–40 MPa). 

Optimization of the thermal and mechanical properties of PP-wollastonite composite drawn fibers was performed based on experiments that were selected through the Box-Behnken method. It was found that the drawing ratio is the most important factor for the increase of tensile strength, while the compatibilizer and the filler content affect this property to a lesser extent. Furthermore, the optimization aiming the simultaneous maximization of mechanical and thermal strength yields results with low desirability values, since the two optimization criteria cannot by simultaneously fulfilled, due to the dual effect of the compatibilizer, i.e., it presents low thermal stability and stretching potential, but, on the same time facilitates the dispersion of the filler and the antioxidant. 

Having in mind that the tensile strength is of primary interest in most applications, a compromise was made by setting as optimization criteria the maximization of tensile strength and a lower limit of the onset decomposition temperature, equal to 300 °C, which can be regarded as satisfactory increase of thermal stability compared to the thermal stability of the neat polymer matrix (the neat PP-antioxidant sample presents onset decomposition temperature of 271 °C). The optimization based on such criteria yields predictions with high desirability values and in very good agreement with verification experiments.

Overall, our results imply that additives, such as antioxidants, combatibilizers, coloring or UV protection agents, which are commonly used in industrial practice and which are not accounted for in most research studies, may have a strong effect on the properties of drawn polymer fibers. Future work may include the development and evaluation of additives specially designed for drawn polymer materials and also the study of other needle-like fillers, which seem to present high potential in polymer fiber applications.

## Figures and Tables

**Table 1:** Materials used and their characteristics.

Material	Abbreviation	Characteristics ^1,2,3,4^	Supplier
Isotactic PP (ECOLEN HZ42Q)	PP	*MFI* = 18 g/10min, *TS* = 33 MPa, *T_m_* = 168–171 °C	Hellenic Petroleum S.A.
Masterbatch with combatibilizer (Bondyram 1001)	MA	PP grafted with maleic anhydride (PP-g-MA). MA content 1%, *MFI* = 100 g/10 min, *T_m_* = 160 °C	Polyram Plastic Industries LTD
Masterbatch with antioxidant (KRITILEN^®^ AO PP9216)	AO	PP with 20.5% wt. antioxidant (combination of phosphite and phenolic types)	Plastika Kritis S.A.
Masterbatch with wollastonite (KRITILEN^®^ D05-00047)	WO	PP with 30% wt. wollastonite of high aspect ratio (*D*_50_ = 3 μm) *MFI* ^5^ = 25 g/10 min	Plastika Kritis S.A.

^1^*MFI*: melt flow index, ^2^*TS*: tensile strength, ^3^*T_m_*: melting point, ^4^*D*_50_: Mass-median-diameter. ^5^ MFI of the PP used for the preparation of the masterbatch and not the MFI of masterbatch itself.

**Table 2:** Prepared composites and their composition.

Composite	Filler Content (%wt.)	Antioxidant Content (%wt.)	Compatibilizer Content ^a^ (%wt)
C0	-	0.82	-
C1	4	0.82	-
C2	-	0.82	15
C3	4	0.82	15
C4	-	0.82	7.5
C5	4	0.82	7.5
C6	2	0.82	-
C7	2	0.82	15
C8	2	0.82	7.5

^a^ On masterbatch base.

**Table 3:** Process parameters values for the fifteen DOE experiments.

ID	Wollastonite Content (%wt.)	Compatibilizer Content ^a^ (%wt.)	λ	Composite ^b^
1	0	0	7	C0
2	4	0	7	C1
3	0	15	7	C2
4	4	15	7	C3
5	0	7.5	5	C4
6	4	7.5	5	C5
7	0	7.5	9	C4
8	4	7.5	9	C5
9	2	0	5	C6
10	2	15	5	C7
11	2	0	9	C6
12	2	15	9	C7
13	2	7.5	7	C8
14	2	7.5	7	C8
15	2	7.5	7	C8

^a^ On mastebatch base, ^b^ see Table 2.

**Table 4:** Experimental results for all the 15 samples.

DOE ID	*EM* (ΜΡa)	*TS* (ΜΡa)	% *EatB*	*T_dec_* (°C)	Δ*H* (J g^−1^)	*T_m_* (°C)
1	1929	296	164	271	86.8	166.7
2	2611	390	178	329	91.8	165.1
3	1791	299	182	350	92.6	167.3
4	1621	291	188	297	86.3	167.3
5	1928	347	214	295	92.6	164.6
6	2016	336	182	300	92.9	166.4
7	2911	521	153	291	95.3	167.6
8	3323	527	150	296	96.9	168.3
9	2111	383	180	304	87.2	164.6
10	1678	310	190	342	98.9	164.9
11	3305	535	148	312	102.1	163.0
12	2921	438	154	298	95.1	161.8
13	2449	360	178	302	96.8	166.9
14	2286	341	188	305	96.4	166.2
15	2203	400	174	305	99.4	164.6

**Table 5:** Tensile test and TGA experimental results compared with theoretical predictions.

	Tensile Strength, *TS*/MPa	Onset Decomposition Temperature, *T_dec_*/°C
Verification Sample	476	302
Optimization prediction	486	300
% Average Absolute Deviation	2.1%	0.7%

## Data Availability

Not applicable.

## Supplementary Materials

The following supporting information can be downloaded at: https://www.mdpi.com/article/10.3390/polym14050924/s1, Table S1: Diameters of two characteristic fiber samples; Figure S1: Optimization based on the combination (overlapping) of contour plots for specified ranges of Δ*H*, *T_dec_* and *TS* assuming a hold (constant) value for the drawing ratio equal to 8.2; Figure S2: Optimization based on the combination (overlapping) of contour plots for specified ranges of Δ*H*, *T_dec_* and *TS* assuming a hold (constant) value for the drawing ratio equal to 7.5; Figure S3: Optimization based on the combination (overlapping) of contour plots for specified ranges of Δ*H*, *T_dec_* and *TS* assuming a hold (constant) value for the filler content equal to 2% wt.; Figure S4: Optimization based on the combination (overlapping) of contour plots for specified ranges of Δ*H*, *T_dec_* and *TS* assuming a hold (constant) value for the compatibilizer content equal to 7.5% wt.; Figure S5: Optimization results targeting maximization of the onset decomposition temperature (*T_dec_*) and the tensile strength (*TS*); Figure S6: Optimization results targeting maximization of tensile strength (*TS*).
