# Supplementary material for: Optimization of Thermal and Mechanical Properties of Polypropylene-Wollastonite Composite Drawn Fibers Based on Surface Response Analysis
Source: Polymers (Basel). 2022 Feb 25;14(5):924. doi: 10.3390/polym14050924 (PMC8912407; doi:10.3390/polym14050924)
Supplement: Supplementary file 1 [file polymers-14-00924-s001.zip › polymers-1597841-supplementary.pdf]

Supplementary Information for

Optimization of Thermal and Mechanical properties of Polypropylene-Wollastonite Composite Drawn Fibers Based on Surface Response Analysis

Konstantinos Leontiadis[1], Costas Tsioptsias[1*], Stavros Messaritakis[2], Aikaterini Terzaki[2], Panagiotis Xidas[3], Kyriakos Mystikos[3], Evangelos Tzimpilis[1] and Ioannis Tsivintzelis[1*]

[1] *Department of Chemical Engineering, Aristotle University of Thessaloniki, University Campus, GR-54124, Thessaloniki, Greece;* leontiad@cheng.auth.gr (K.L.); tzimpi@auth.gr (E.T.)

[2] *Plastika Kritis S.A., R Street, Industrial Area of Heraklion, GR-71408, Heraklion, Crete, Greece;* messaritakis@plastikakritis.com (S.M.); terzaki@plastikakritis.com (A.T.)

[3] *Thrace Nonwovens & Geosynthetics S.A., Magiko, GR-67100, Xanthi, Greece;* pxidas@thraceplastics.gr (P.X.); kmystikos@thraceplastics.gr (K.M.)

* Correspondence: ktsiopts@gmail.com (C.T.); tioannis@cheng.auth.gr (I.T.)

Table S1. Diameters of two characteristic fiber samples

Repetition	Diameter, mm	
	DOE 3	DOE 11
1	0.232	0.147
2	0.230	0.144
3	0.236	0.144
4	0.239	0.148
5	0.223	0.154
6	0.225	0.157
7	0.235	0.160
8	0.232	0.155
9	0.234	0.173
10	0.242	0.174
**Average value ± standard deviation**	**0.23 ± 0.01**	**0.16 ± 0.01**

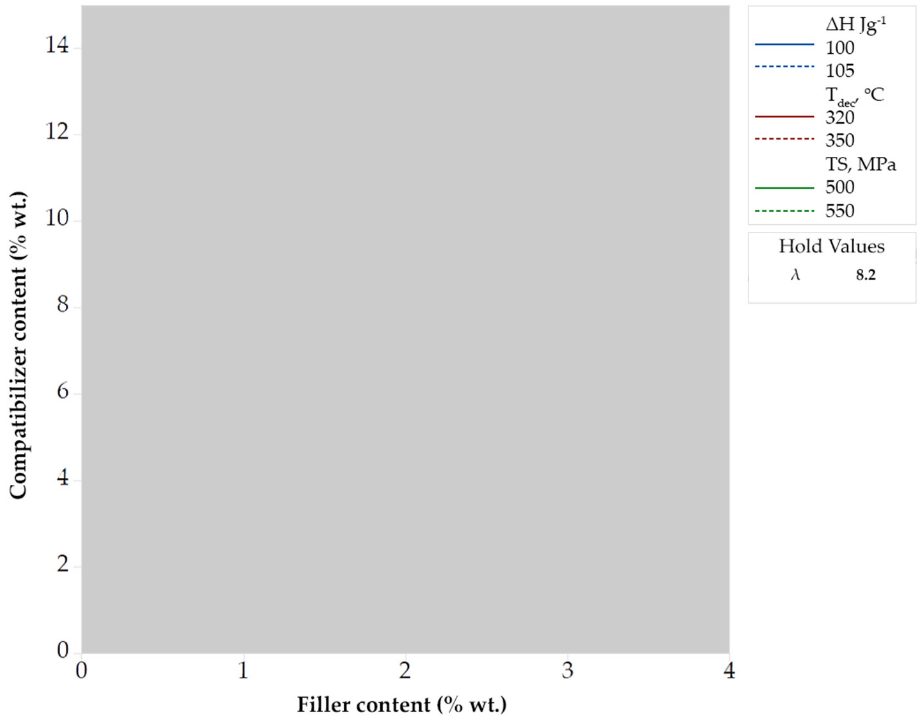

Figure S1. Optimization based on the combination (overlapping) of contour plots for specified ranges of ΔH, T_{dec} and TS assuming a hold (constant) value for the drawing ratio equal to 8.2.

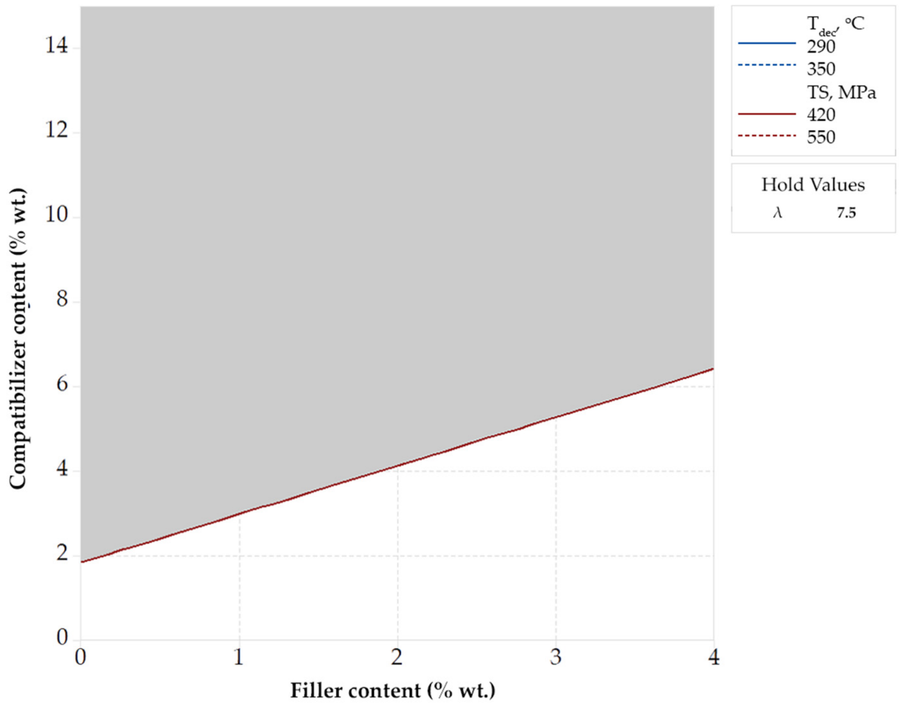

Figure S2. Optimization based on the combination (overlapping) of contour plots for specified ranges of ΔH, T_{dec} and TS assuming a hold (constant) value for the drawing ratio equal to 7.5.

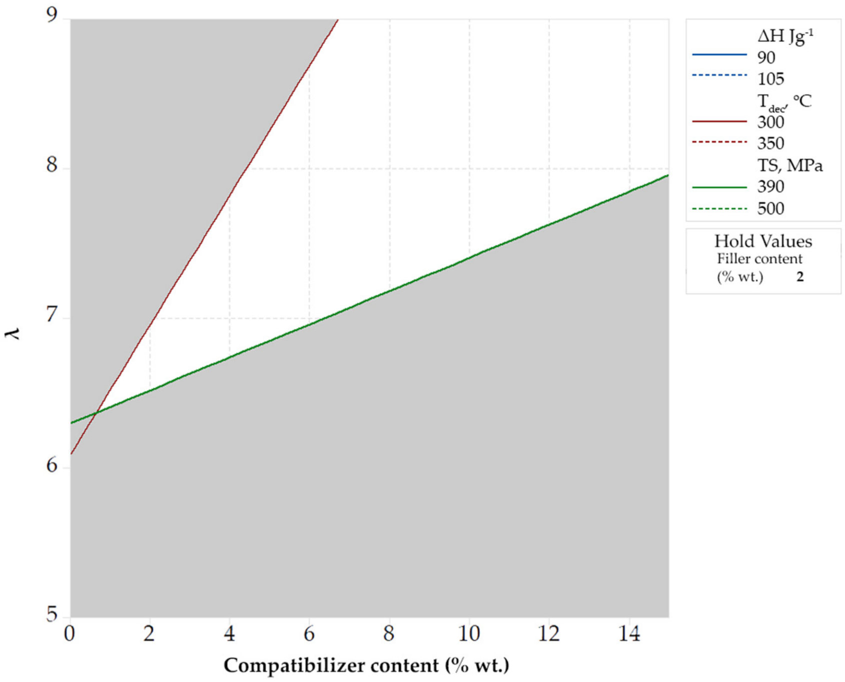

Figure S3. Optimization based on the combination (overlapping) of contour plots for specified ranges of ΔH, T_{dec} and TS assuming a hold (constant) value for the filler content equal to 2% wt.

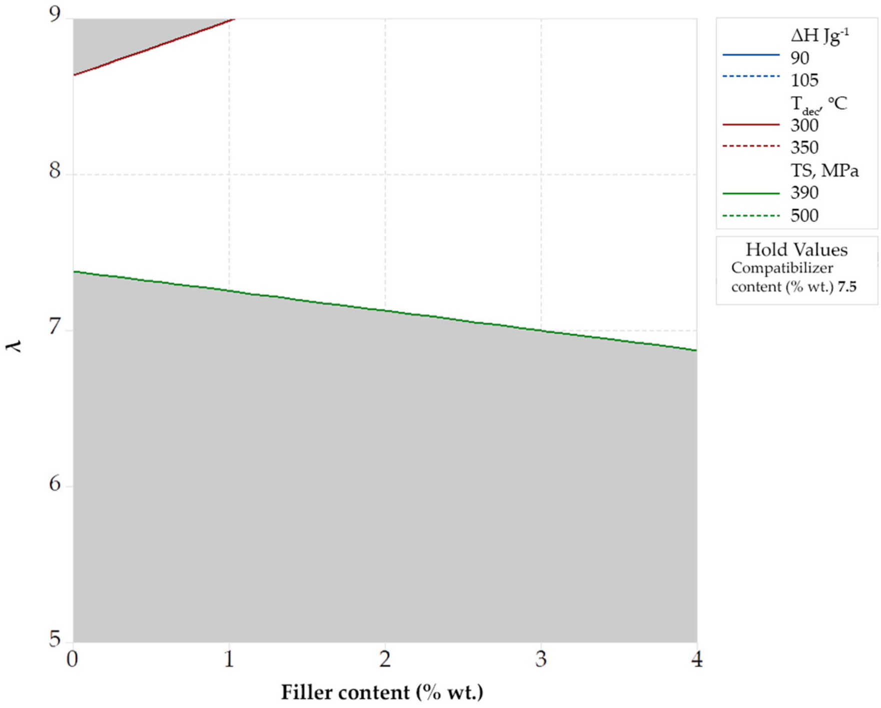

Figure S4. Optimization based on the combination (overlapping) of contour plots for specified ranges of ΔH, T_{dec} and TS assuming a hold (constant) value for the compatibilizer content equal to 7.5% wt.

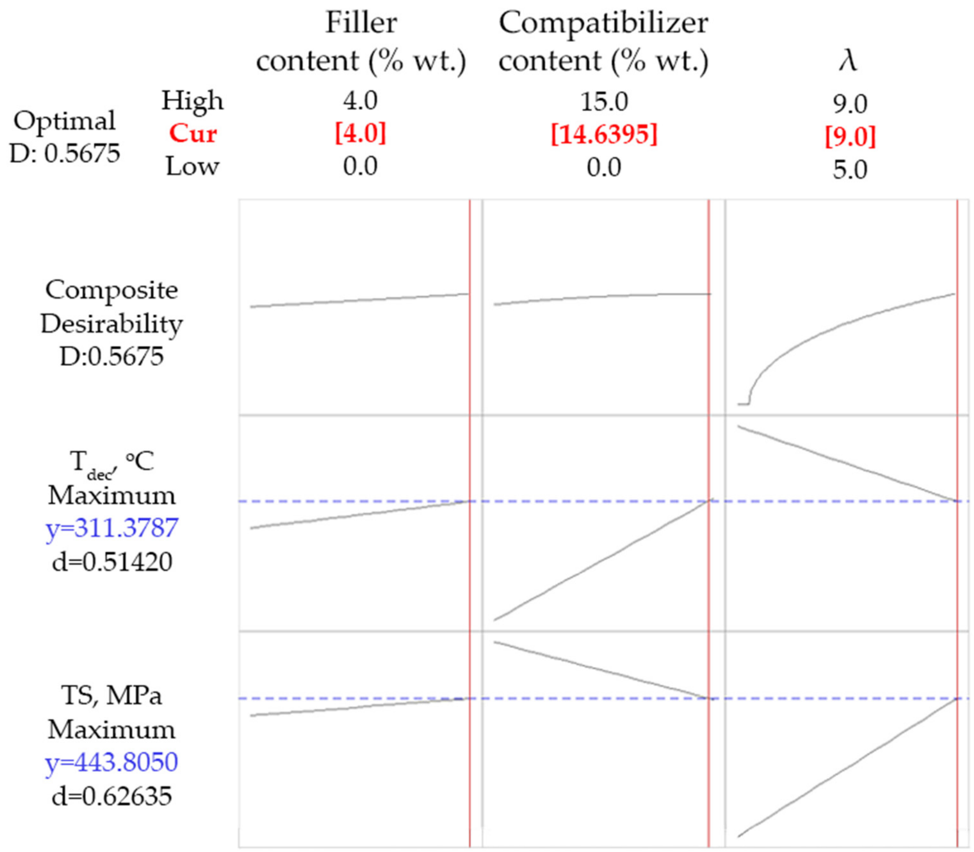

Figure S5. Optimization results targeting maximization of the onset decomposition temperature (Tdec) and the tensile strength (TS).

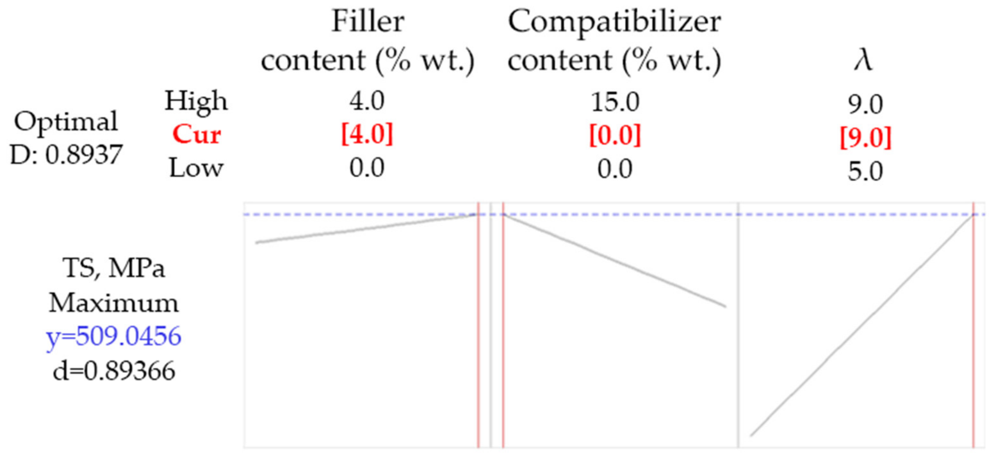

Figure S6. Optimization results targeting maximization of tensile strength (TS).
